# Role of Integrin β1 in the progression and chemo-resistance of esophageal squamous cell carcinoma

**DOI:** 10.7150/jca.68647

**Published:** 2022-03-28

**Authors:** Ying-Hua Xie, Li-Qiang Ran, Zhi-Yong Wu, Chun Sun, Xiu-E Xu, Hai-Ying Zou, Wang-Kai Fang, Jian-Jun Xie

**Affiliations:** 1 Department of Biochemistry and Molecular Biology, Shantou University Medical College, Shantou, China.; 2 Department of Surgical Oncology, Shantou Central Hospital, Affiliated Shantou Hospital of Sun Yat-sen University, Shantou, China.; 3 Institute of Oncologic Pathology, Shantou University Medical College, Shantou, China.

**Keywords:** Integrin α5β1, Biomarker, Prognosis, Chemo-resistance, ESCC

## Abstract

**Objective:** Integrins have been shown to play an important role in the tumorigenesis of many cancers. In this work, we aimed to explore the expression and clinical value of Integrin α5β1 in esophageal squamous cell carcinoma (ESCC), and the effect of integrin β1 on the development and chemo-resistance of ESCC cells.

**Methods:** The expression profiling of integrins was analyzed in the mRNA expression dataset of ESCC. The expression of Integrin α5β1 in 278 cases of ESCC tissues and 62 cases of paracancerous tissues was detected by immunohistochemistry (IHC). The association between the expression of Integrin α5β1 and the survival of ESCC patients was analyzed by Kaplan-Meier analysis. The effect of Integrin β1 on the proliferation, migration, and invasion of ESCC cells was examined by MTS, Transwell migration, and Transwell invasion assay. The effect of Integrin β1 and L1 cell adhesion molecule (L1CAM) on cisplatin resistance was detected by MTS and the signal pathways involved were analyzed by Western blotting.

**Results:** Integrin β1 and Integrin α5 were significantly up-regulated in ESCC. High expression of Integrin β1 was also related to worse overall survival of ESCC patients and patients with low levels of both Integrin β1 and Integrin α5 showed the shortest survival. Results of IHC revealed that Integrin α5β1 was up-regulated in ESCC and its high expression was associated with poor prognosis and could serve as an independent prognostic factor. siRNA-mediated Integrin β1 silencing or antibody blocking restrained the proliferation, migration, and invasion of ESCC cells. Simultaneous knockdown of Integrin β1 and L1CAM reduced the cisplatin resistance of ESCC cells. Further studies showed that knockdown of Integrin β1 and L1CAM suppressed the activity of Akt signaling with or without cisplatin treatment. Moreover, dual high expression of Integrin β1 and L1CAM was related to worse overall survival of ESCC patients treated with preoperative chemotherapy.

**Conclusion:** Integrin α5β1 was up-regulated in ESCC and could be used as a new prognostic indicator for ESCC patients. In addition, Integrin β1 was involved in the proliferation, invasion, and chemo-resistance of ESCC cells.

## Introduction

Esophageal cancer is a common malignant tumor. It ranks seventh in the incidence rate of all cancers worldwide and sixth in the mortality rate^1^. The highest regional incidence rates are observed mainly in Eastern Asia, in part because of the large burden in China, followed by Southern Africa and Eastern Africa. There are two main histological subtypes of esophageal cancer, esophageal squamous cell carcinoma (ESCC) and adenocarcinoma (EAC). Worldwide, more than 90% of esophageal cancer is ESCC^2^. Traditional multidisciplinary therapy, including radiotherapy, chemotherapy, and surgery, as well as novel therapeutic strategies such as cancer vaccines and immune checkpoint inhibitors, have been applied to the treatment of ESCC^3^-^6^. However, the 5-year survival rate for ESCC patients in the United States was 20% in 2010-2016^7^. In China, the 5-year survival rate was 32.3% in rural areas and 18.1% in urban areas in 2012-2015^8^. Therefore, it is urgent to develop new therapeutic strategies, such as targeted molecular therapy, to increase the survival chances against ESCC.

Integrins are a superfamily of alpha-beta heterodimeric receptors, which play an essential role in cell adhesion by binding to extracellular matrix ligands, cell-surface ligands, and soluble ligands^9^. In humans, the family consists of 18 alpha and 8 beta subunits that combine to form at least 24 distinct types of αβ integrin heterodimers. As the only known α5 integrin heterodimer, Integrin α5β1 is overexpressed in cervical cancer^10^, ovarian cancer^11^, ^12^, and recurrence colorectal cancer^13^, indicating a poor prognosis. Integrin α5β1 is up-regulated on the luminal surface of tumor vessels and plays a key role in angiogenesis, leading to tumor growth^14^, ^15^. Integrin α5β1 also plays a critical role in the migration and invasion of tumor cells by enhancing cell adhesion, reprograming the actin cytoskeleton, and regulating the expression and/or activity of matrix metalloproteinases (MMPs)^16^-^21^. In addition, mounting studies have implicated that Integrin α5β1 contributed to the chemo-resistance and radio-resistance of tumor cells. Cell adhesion-mediated drug resistance (CAM-DR), which is induced by Integrin α5β1-mediated adhesion, protects human myeloma cells from apoptosis induced by DNA damage agents and gamma-irradiation^22^. Besides, binding of fibronectin to Integrin α5β1 can increase the resistance of head and neck squamous cell carcinoma (HNSCC) cells to paclitaxel-mediated apoptosis^23^.

In ESCC, the co-overexpression of TM4SF5 and Integrin β1 indicated a poor prognosis^24^. Overexpression of Integrin β1 was associated with docetaxel resistance in ESCC cells^25^. Down-regulation of AQP3 expression inhibited the expression of Integrin α5, β1 and cell adhesion ability, while the phosphorylation levels of FAK, ERK and MAPK decreased, and cisplatin resistance weakened^26^. Another study showed that high expression of Integrin β1 promoted metastasis and confers cisplatin resistance to ESCC^27^. Here, we investigated the expression of Integrin α5β1 in clinical samples of ESCC, as well as the effects of Integrin β1 on proliferation, migration, invasion, and cisplatin resistance of ESCC cells, evaluating its potential as a molecular therapeutic target for ESCC.

## Materials and Methods

### Clinical Specimen Collection

The data set of formalin-fixed, paraffin-embedded tissue specimens were obtained from ESCC patients undergoing curative resection at the Shantou Central Hospital from 2008 to 2014, including 278 cases of esophageal carcinoma and 62 cases of paracancerous tissue. All specimens were confirmed as ESCC by pathologists in the Clinical Pathology Department of the hospital. Ethical approval was obtained from the ethical committee of Shantou Central Hospital and the ethical committee of the Medical College of Shantou University. Only resected samples from surgical patients with written informed consent were included.

### Immunohistochemistry

Tissue microarrays (TMA), immunohistochemistry (IHC), and evaluation of IHC variables were performed as described previously^28^. Briefly, TMA with one-millimeter cores was constructed from representative regions of each tissue block which were singled out of hematoxylin and eosin-stained sections in advance. The cores were arrayed into a new paraffin block. For immunohistochemistry, these microarrays were cut into serial 4-μm thick sections, and routine dewaxing, hydration, and antigen repair were performed. TMA sections were incubated with anti-Integrin α5β1 antibody (MAB1969, Millipore, 1:50) overnight at 4°C, and then subjected to immunostaining in the Polymer Detection System (PV-9000, ZSGB-BIO) and the DAB Kit (ZLI-9017, ZSGB-BIO). Subsequently, slides were counterstained with hematoxylin, dehydrated, and mounted. The negative controls were treated with phosphate buffered saline (PBS) instead of primary antibody. The score of immunohistochemical staining was mainly based on the intensity of staining and the number of tumor cells showing unequivocal positive reaction. Positive reaction was defined as the reaction of brown signal on cytoplasm and cell membrane. The intensity of staining: 0, no staining; 1, weak staining; 2, moderate staining; and 3, strong staining. The number of tumor cells with positive staining: 0, less than 5% of tumor cells showed positive staining; 1, 5-25% of tumor cells showed positive staining; 2, 26-50% of tumor cells showed positive staining; 3, 51-75% of tumor cells were positive; 4, more than 76% of tumor cells showed positive staining. Each section was independently evaluated by two histopathologists who had no prior knowledge of patient data. The two scores were multiplied as the comprehensive score. To facilitate the follow-up statistical analysis, the cases were divided into two groups: the low expression group with a comprehensive score of less than 5; the high expression group with a comprehensive score of 5-12.

### Cell Culture and transfection

Details of cell lines used in this study have been described previously^29^. The KYSE150 and KYSE180 ESCC cell lines were cultured in Roswell Park Memorial Institute (RPMI) 1640 medium (11875119, Invitrogen Life Technologies, USA) with 10% fetal bovine serum (10099141C, Invitrogen). All cells were incubated at 37°C in a humidified atmosphere containing 5% CO2. Cells were tested to ensure they were mycoplasma-negative. Integrin β1 was knockdown by siRNA transfection or lentiviral infection. 48 hrs after virus transfection, stably transfected cell strains were screened with puromycin (500ng/mL), and the transfection efficiency was verified by Western blot assay. siRNA (Qiagen, Germany) and shRNA (Hanbio, China) targeting Integrin β1 contained the following sequence: 5'- ACAGATGAAGTTAACAGTGAA -3'.

### RNA Extraction and qRT-PCR analysis

Total RNA from ESCC cells was isolated with TRIzol (15596018, Invitrogen) as per the manufacturer's instructions, and the concentration was determined by a Nanodrop 2000 (Thermo Fisher Scientific). One microgram of total RNA was reverse transcribed into cDNA by PrimeScript™ RT reagent Kit with gDNA Eraser (RR047A, Takara Bio Inc.) according to the manufacturer's protocol. Quantitative real-time PCR (qRT-PCR) was performed by SYBR® Premix Ex Taq™ (Tli RNaseH Plus) (RR420B, Takara Bio Inc.) using a 7500 Real-Time PCR System (Applied Biosystems). Primer pairs for target genes used in the PCR assay were: Integrin β1 Forward: 5'-GAGGAAATGGTGTTTGCAAGTG-3', Reverse: 5'-TATGCTCAGCACAGACACCAA-3'; and β-actin Forward: 5'-CAACTGGGACGACATGGAGAAA-3', Reverse: 5'-GATAGCAACGTACATGGCTGGG-3'. β-actin was measured as an internal control and used for normalization. All experiments were replicated at least three times with n = 3 samples per experiment.

### Western blotting

Total cell lysates were prepared from confluent cultures in RIPA buffer (P0110, Maygene). Blots were incubated with primary antibodies against Integrin β1 (610468, BD Biosciences, 1:800), L1CAM (Ab24345, Abcam, 1:1000), pAKT (Thr308) (2965, CST, 1:1000), AKT (4691, CST, 1:1000), pSTAT3 (Tyr705) (4113, CST, 1:1000), STAT3 (12640, CST, 1:1000), α-Tubulin (2144, CST, 1:100) and β-actin (sc-47778, Santa Cruz, 1:1000) at 4 ºC overnight. The secondary antibody (sc-2005, Santa Cruz, 1:5000) was performed to incubate with membranes at room temperature for 1hour (h). Signals were detected, with luminol reagent, using an imaging system (FluorChem TM8900, Alpha Innotech).

### Cell Proliferation Assay

Integrin β1 was knockdown by siRNA or shRNA or blocked by peptide JB1A (MAB1965, Millipore, 1:100) in ESCC cells. Cell proliferation was identified by MTS. In brief, the cells in the logarithmic growth phase were plated in 96-well plates at 6000 per well. 20 μL of the MTS reagent (G3580, Promega) was added to each well and the quantity of formazan product was measured by absorbance at 490nm after 2 hours of culture. Experiments were repeated three times in each group.

### Transwell Migration Assay and Transwell Invasion Assay

Migration and invasion assays were performed with a chemotaxis chamber (353097, BD Biosciences). The cells were starved in serum-free medium for 12 h before being harvested. A total of 5x10^4^ cells (~400 μl) were plated in the upper chambers and the lower chambers were filled with 500 μl 1640 media supplied with 10% FBS. Following 48h of free migration, the membrane was fixed with fixative and stained with hematoxylin at room temperature for 30 min. Cells in the bottom of the chamber were then counted under a microscope (magnification, ×200). For the invasion assay, the membrane of the chamber was coated with a Matrigel solution (354234, Corning Inc.) 1 h before the experiment in a 37˚C incubator. Cells were allowed to invade for 48h before staining by hematoxylin. All procedures were repeated in triplicate.

### Cisplatin resistance test

The cisplatin resistance of ESCC cell lines was detected by MTS. The cells were plated in 96-well plates at 7000 cells per well for 12h and then were exposed to the indicated concentrations of cisplatin (S1166, Selleck). Cell viability was detected at the indicated time by adding 20 μL of the MTS reagent per well and the quantity of formazan product was measured by absorbance at 490nm after 2 hours of culture. For signaling pathways analysis, the cells were plated in 12-well plates at 2×10^5^ cells per well for 12h and then were exposed to 4 μM cisplatin or normal medium for 24h. The cell lysates were collected by RIPA lysis buffer (P0110, Maygene) for Western blot.

### Statistical analysis

All analyses were performed on IBM SPSS 21.0 Software and GraphPad Prism 7.0 Software. The differences between groups were determined with unpaired t test. Kaplan-Meier methods and logrank test were used to evaluate the overall survival curve. The Cox proportional hazard model was performed for both univariate and multivariate models to estimate hazard ratios (HRs) and confidence intervals (CIs). *P* < 0.05 was judged as statistically significant.

## Results

### Expression profiling of integrins in ESCC

**Figure 1:**
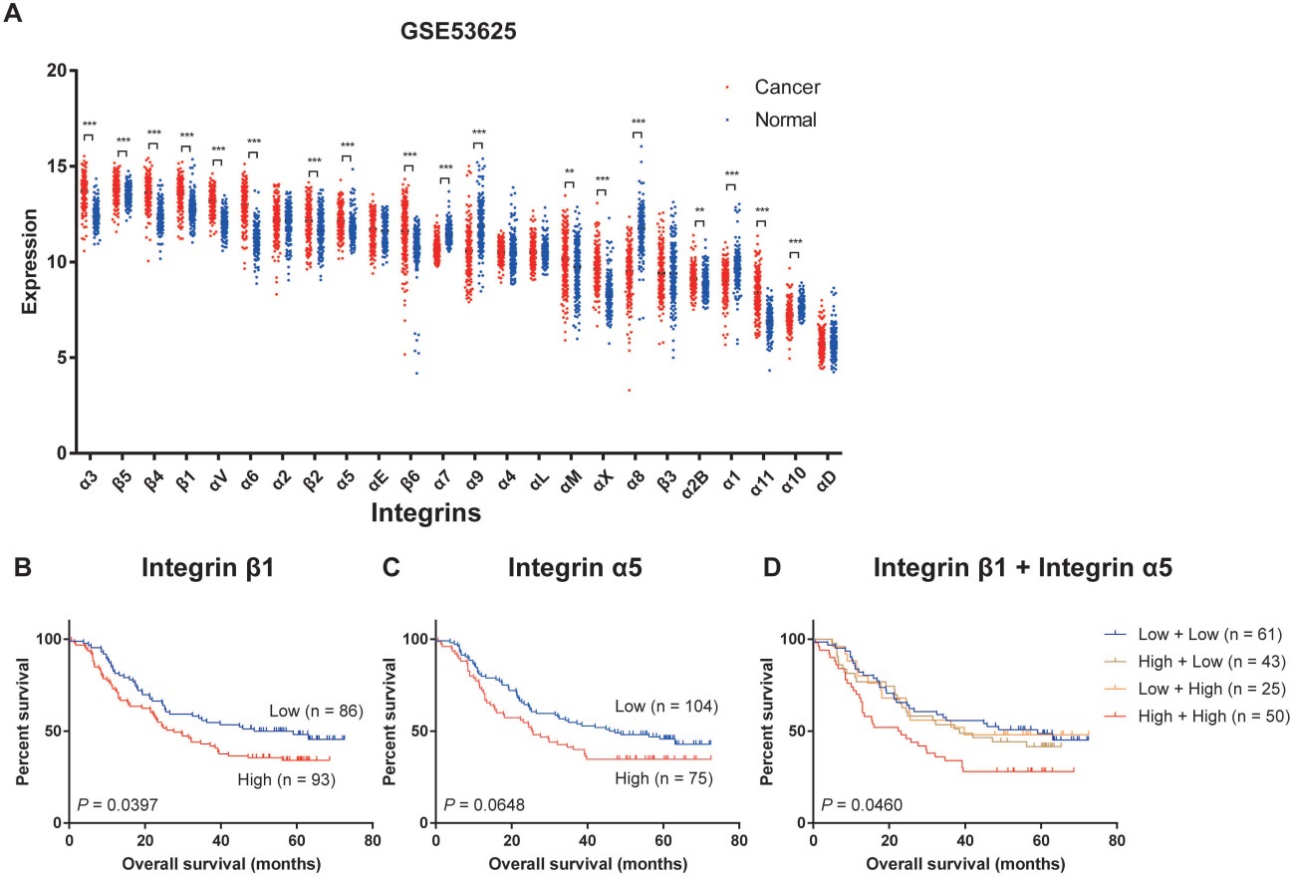
** Expression profiling of integrins in ESCC.** (A) The integrins mRNA expression profile of paired cancer and adjacent normal tissues from 179 ESCC patients (data extracted from GSE53625 dataset in GEO database). Mean ± SD. Multiple t-tests. **, *P* < 0.01, ***, *P* < 0.001. (B) Kaplan-Meier estimates of the overall survival by Integrin β1 expression in ESCC samples. (C) Kaplan-Meier estimates of the overall survival by Integrin α5 expression in ESCC samples. (D) Kaplan-Meier estimates of the overall survival by Integrin β1 and Integrin α5 expression in ESCC samples. A *P* value of less than 0.05 was considered statistically significant.

We firstly analyzed the transcriptional expression profiling of integrins in the GSE53625 dataset, the mRNA expression profile of paired cancer and adjacent normal tissues from 179 ESCC patients, and relevant clinical information from the Gene Expression Omnibus (GEO) database^30^. Twenty-four members of the integrin family could be detected in ESCC tissues and adjacent normal tissues. The expression levels of the different integrins in ESCC tissues varied considerably with Integrin α3, β4, β5, and β1 displaying the highest expression levels and Integrin α1, α11, α10, and αD showing the lowest levels (Figure 1A). When compared with the normal tissues, Integrin α3, β4, β5, β1, αV, α6, β2, α5, β6, αM, αX, α2B, and α11 were significantly up-regulated in ESCC, and expression levels of Integrin α7, α9, α8, α1, and α10 were decreased (Figure 1A). Similar results had been verified in The Cancer Genome Atlas (TCGA) and The Genotype-Tissue Expression (GTEx) datasets and GSE53624 dataset from GEO databases (Figure S1 and S2A)^30^. In all three datasets, Integrin α3, β4, β1, αV, α6, β2 and α11 showed high expression in tumor tissues, while Integrin α7 and α10 was low expressed in tumor tissues (Figure S2B), suggesting that the differential expression of these integrins in esophageal cancer was universal.

Next, we analyzed the overall survival in patients grouped according to the expression level of a specific integrin. This revealed that patients which express high Integrin β1 had a significantly shorter overall survival compared to patients with low expression of Integrin β1 (Figure 1B). High expression of Integrin α5 was also related to worse overall survival of ESCC patients, but the difference was not statistically significant (Figure 1C). In addition, patients with high expression of both Integrin β1 and Integrin α5 had the worst overall survival (Figure 1D). No association was found between any other integrin and the survival of ESCC patients.

### Integrin α5β1 was up-regulated in ESCC and associated with poor prognosis

**Figure 2:**
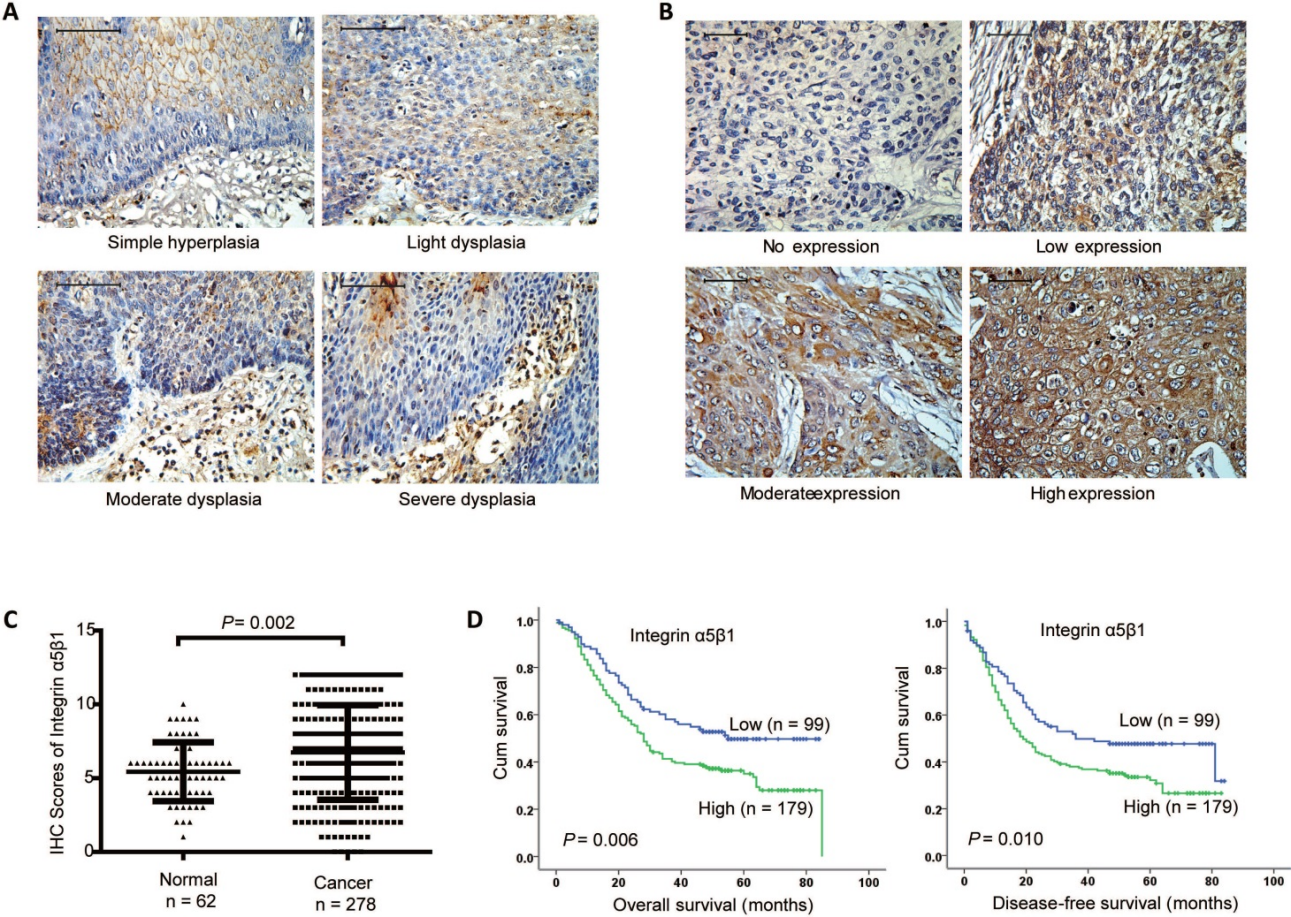
** Integrin α5β1 was up-regulated in ESCC and associated with poor prognosis.** (A) Representative IHC staining images of Integrin α5β1 in normal esophageal epithelium samples. (B) Representative IHC staining images of Integrin α5β1 in ESCC tissues. (C) Scoring analysis of Integrin α5β1 in normal esophageal tissues and ESCC tissues. (D) Kaplan-Meier estimates of the overall survival (left) and disease-free survival (right) by Integrin α5β1 expression in ESCC samples. A *P* value of less than 0.05 was considered statistically significant. Bar, 50 μm.

To investigate the clinical role of Integrin α5β1, the protein heterodimer of Integrin β1 and Integrin α5 in ESCC, IHC was performed to examine the protein expression of Integrin α5β1 in 62 paracancerous tissues and 278 ESCC tissues. In paracancerous tissues, Integrin α5β1 was mainly expressed in cell membrane and cytoplasm, but less in stratum basale than other stratums (Figure 2A). In the cancerous tissues, Integrin α5β1 was also localized in the cell membrane and cytoplasm, and the positive staining was significantly increased (Figure 2B). Scoring analysis revealed that Integrin α5β1 expression was significantly up-regulated in ESCC by comparing to the NCMT (Figure 1C, *P* = 0.002). Kaplan-Meier analysis suggested the high Integrin α5β1expression was significantly correlated with poorer overall survival (*P* = 0.006) and decrease-free survival (*P* = 0.010) of ESCC patients (Figure 2D). Further study showed that there was no significant correlation between the expression of Integrin α5β1 and the clinicopathological characteristics in ESCC (Table 2). In multivariate analysis, Integrin α5β1 was a significant predictor of worse overall survival (Table 3, HR 1.592, 95% CI 1.138-2.228, *P* = 0.007) and disease-free survival (Table 4, HR 1.504, 95% CI 1.086-2.083, *P* = 0.014), suggesting that Integrin α5β1 was an independent prognostic factor for ESCC patients.

### Integrin β1 promoted proliferation, migration, and invasion of ESCC cells

**Figure 3:**
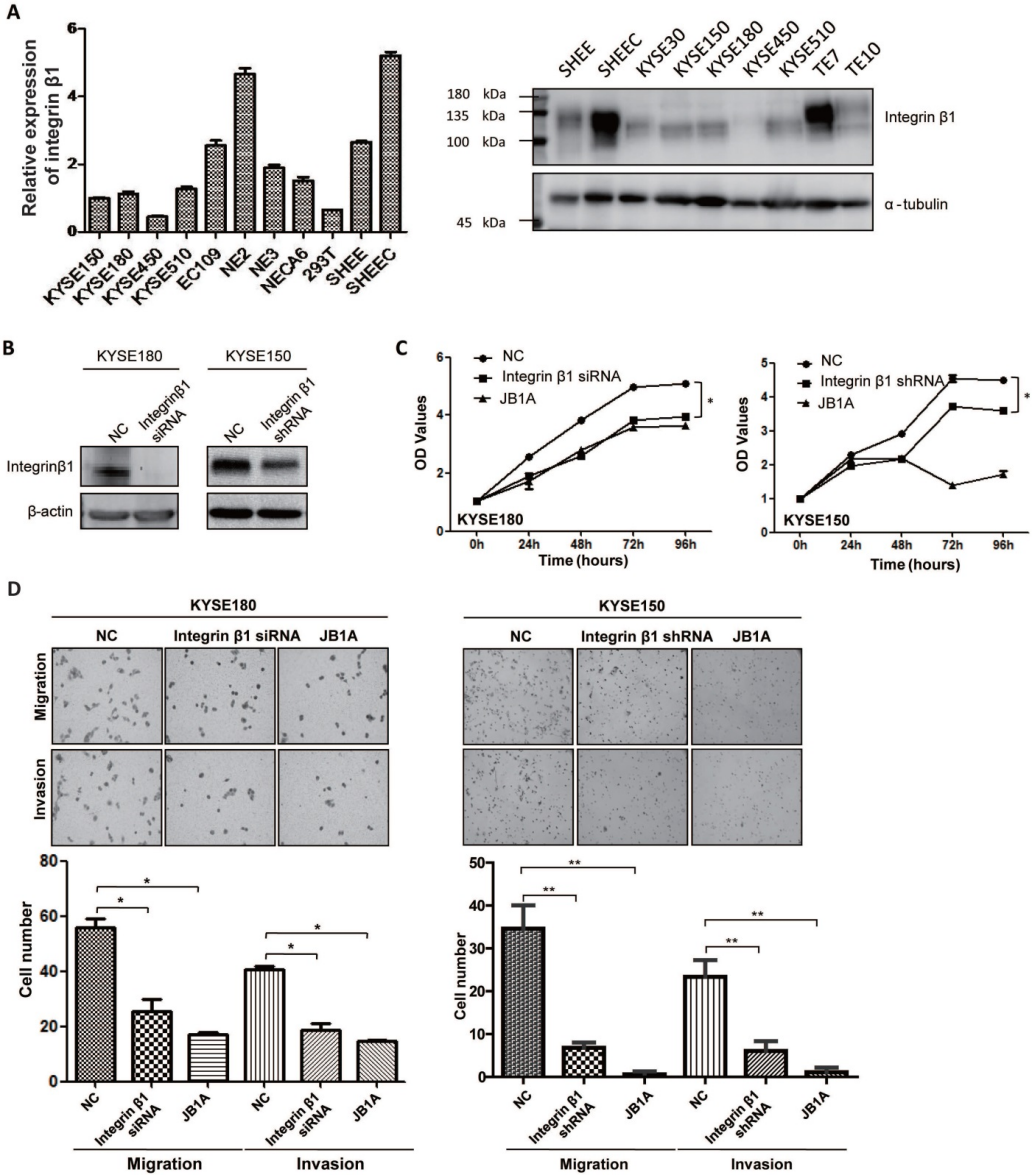
** Integrin β1 silencing suppressed the proliferation, migration, and invasion of ESCC cells.** (A) The expression of Integrin β1 protein in ESCC cell lines (KYSE30, KYSE150, KYSE180, KYSE450, KYSE510, TE7, TE10, EC109, SHEEC), immortalized esophageal epithelial cell lines (NE2, NE3, NECA6, SHEE) and 293T cell line by qRT-PCR (left) and Western blot (right). (B) KYSE180 (left) and KYSE150 (right) were transfected with negative control siRNA/shRNA (NC) or anti-Integrin β1 siRNA/shRNA. The knockdown of Integrin β1 was evaluated by Western blotting. β-actin was served as a loading control. (C) MTS assay was conducted to measure the proliferation of ESCC cells after Integrin β1 knockdown by siRNA/shRNA or blocked by JB1A, a blocking antibody of Integrin β1. (D) Transwell Migration Assays and Transwell Invasion Assays were performed to measure the migration and invasion of ESCC cells after Integrin β1 knockdown by siRNA/shRNA or blocked by JB1A. The independent sample t-test was used to determine the significance of differences between groups and data was obtained in at least three independent experiments in (C) and (D). Average values are given ± SD. *, *P* < 0.05; **, *P* < 0.01.

Next, we tried to explore the effect of Integrin β1 on the tumorigenesis of ESCC. The mRNA and protein expression levels of Integrin β1 in ESCC cell lines were shown in Figure 3A. KYSE150 and KYSE180, two ESCC cell lines, with a relatively intermediate expression of Integrin β1, were used for further research. Integrin β1 was knocked down in KYSE150 and KYSE180 cell lines with siRNA or shRNA and the knockdown efficiency was validated by Western blotting (Figure 3B). MTS assay showed that both down-regulating Integrin β1 by siRNA or shRNA and inhibiting its function by antibody blocking (JB1A) significantly decreased the proliferation of ESCC cells (Figure 3C). It has been reported that Integrin β1 promotes migration and invasion in ESCC lines 30D and TE10^27^, and we confirmed this result in ESCC lines KYSE150 and KYSE180 by downregulating or blocking Integrin β1 (Figure 3D). Another Integrin β1 siRNA has been used to verify the above experiments to eliminate the off target effect (Figure S3A-C). These results indicate that Integrin β1 plays a crucial role in maintaining the malignant phenotypes of ESCC cells.

### Integrin β1 and L1CAM synergistically enhanced the chemo-resistance of ESCC cells

**Figure 4:**
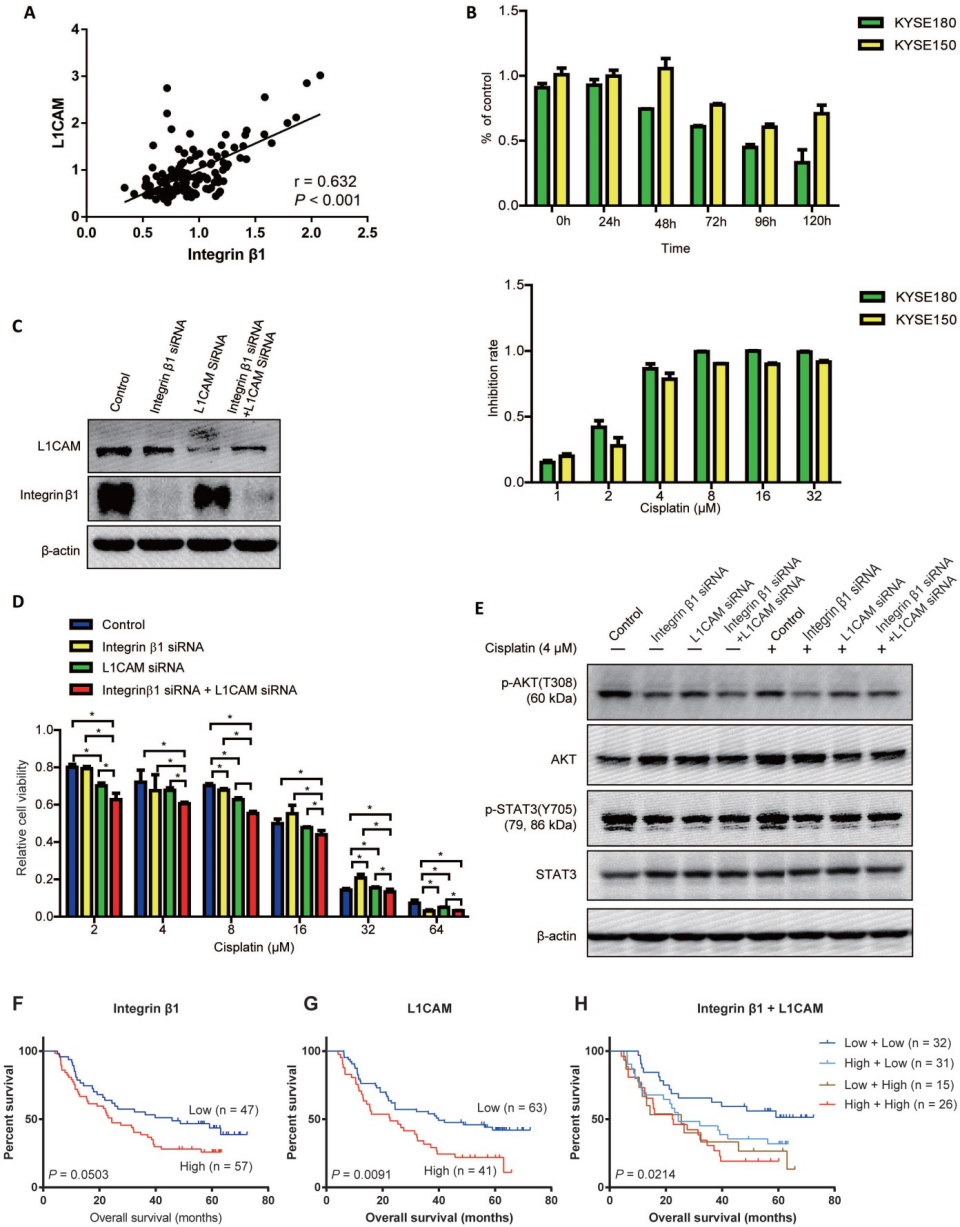
** Integrin β1 and L1CAM synergistically enhanced the cisplatin resistance of ESCC cells by suppressing AKT signaling.** (A) Correlation analysis of Integrin β1 and L1CAM expression in esophageal carcinoma. The proteome datasets were obtained from PRIDE database (accession number PXD021701). (B) The cisplatin resistance of ESCC cell lines was detected by MTS. Above: KYSE180 and KYSE150 cells were exposed to cisplatin (4 μM), and cell viability was detected at the indicated time. Below: KYSE180 and KYSE150 cells were exposed to the indicated concentrations of cisplatin for 72 hours, and then the cell viability was detected. (C) The expression of Integrin β1 and L1CAM in KYSE150 was detected by Western blotting. KYSE150 was transfected with integrin β1 and/or L1CAM siRNA. After 36 hours, some cells were used for the cisplatin resistance test, and the remaining cells were cultured for 12 hours to collect cell lysates, followed by Western blot. (D) After Integrin β1 and/or L1CAM silence by siRNA, KYSE150 was exposed to the indicated concentrations of cisplatin for 72 hours, and then the cell viability was detected by MTS. The independent sample t-test was used to determine the significance of differences between groups. (E) The signaling pathways involved in the Integrin β1/L1CAM-mediated cisplatin resistance were detected by Western blotting. After Integrin β1 and/or L1CAM silence by siRNA, KYSE150 was cultured in medium with or without 4 μM for 24 hours, and then the cell lysates were collected for Western blotting. Data were obtained in at least three independent experiments in (C), (D), and (E). Average values are given ± SD. *, *P* < 0.05. (F-H) The associations between the expression of Integrin β1 or/and L1CAM with the overall survival of ESCC patients treated with preoperative chemotherapy were determined.

Our previous studies have found that L1 cell adhesion molecule (L1CAM), which was high-expressed in ESCC tissues, interacted with Integrin β1 and upregulated the expression of the cytoskeletal protein ezrin via activating integrin β1/MAPK/ERK/AP1 signaling, leading to the malignant phenotypes of ESCC cells 31. Here, based on the proteomic analysis of 124 esophageal cancer tissues^32^, we found that there was a significant positive correlation between the protein levels of L1CAM and Integrin β1 in ESCC (Figure 4A).

It has been reported that Integrin α5 was indispensable for L1CAM mediated chemo-resistance in pancreatic adenocarcinoma cells^33^. To investigate whether Integrin β1 contributed to L1CAM mediated chemoresistance in ESCC, cisplatin was used to construct a chemotherapy model and followed down-regulation of Integrin β1 and L1CAM. The results showed that the optimal reaction time of cisplatin was 72 hours, and the half-maximal inhibitory concentration of cisplatin was 2.411μM and 2.956μM in KYSE180 and KYSE150, respectively (Figure 4B and Table 5). L1CAM knockdown significantly reduced the cisplatin resistance of ESCC cells, while Integrin β1 knockdown had no significant inhibitory effect on cisplatin resistance (Figure 4C and D). Interestingly, the inhibition of cisplatin resistance by simultaneous deletion of Integrin β1 and L1CAM was more obvious than that by L1CAM deletion alone, suggesting that there was a synergistic effect between Integrin β1 and L1CAM in cisplatin resistance (Figure 4D). Further studies showed that knockdown of Integrin β1 or L1CAM alone could inhibit the phosphorylation of AKT in ESCC cells with or without cisplatin treatment, and in the cells with simultaneous knockdown of Integrin β1 and L1CAM, the inhibition effect was more significant (Figure 4E). However, knockdown of Integrin β1 or L1CAM did not affect STAT3 phosphorylation. These results demonstrated that Integrin β1 and L1CAM synergistically enhanced the chemo-resistance of ESCC cells, at least in part, via the AKT signaling pathway.

Finally, the associations between the expression of Integrin β1 or/and L1CAM with the overall survival of ESCC patients treated with preoperative chemotherapy were determined. No significant association was found between Integrin β1 expression and the survival (*P* = 0.0503, Figure 4F) while higher L1CAM level was related to poorer survival of ESCC patients (*P* = 0.0091, Figure 4G). Moreover, patients whose Integrin β1 and L1CAM were both elevated showed the shortest survival time (*P* = 0.0214, Figure 4H).

## Discussion

As a common malignant tumor, ESCC is in the dilemma of no chemoradiotherapy-sensitive molecular markers and no prognostic molecular markers. It is urgent to explore the development process of ESCC and find specific biomarkers or therapeutic molecular targets. Here, by analyzing the expression profiling of integrins in ESCC, we found that the expression of Integrin α5β1 in ESCC was higher than that in normal esophageal tissue. The high expression of Integrin α5β1 was associated with poor prognosis and could be used as an independent prognostic factor for ESCC patients. These data suggested a potential role of Integrin α5β1 in the treatment of ESCC.

A wide variety of integrins contribute to tumor progression. As many solid tumors originate from epithelial cells, the integrins expressed by epithelial cells (including a6β4, a6β1, αvβ5, a2β1, and a3β1) are generally retained in the tumor, though expression levels may be altered^34^. Several integrins such as Integrin β6, α6, and α11, are up-regulated in ESCC^35^-^37^. However, the expression characteristics of the integrin family in ESCC were unknown. In this study, a comprehensive analysis was performed to determine the transcriptional expression profiling of integrins in ESCC tissues. Certain integrins (Integrin α3, β4, et al.) were over-expressed in ESCC tissues compared to normal tissues while levels of integrins such as Integrin α8 and α9 were down-regulated. Further analysis showed that Integrin β1 and α5 might be associated with the survival of ESCC patients, suggesting that those integrins might play crucial roles in the progression of ESCC.

The tumor microenvironment is a complex meshwork of extracellular matrix (ECM) macromolecules, which harbors cancer cells that interact with surrounding cells through the lymphatic and circulatory to influence tumorigenesis. ECM stiffness and cell tension promote focal adhesion assembly. Proteins that contain the Arg-Gly-Asp (RGD) attachment site, together with the integrins that serve as receptors for them, constitute a major recognition system for cell adhesion. Integrin α5β1 is one of the most inportant receptors recognizing the RGD peptide motifs and Integrin β1 ligation and signaling is essential for tension-dependent tumor invasion and metastasis^38^. The invasion of a premalignant epithelium into a stiffened ECM could be repressed by inhibiting Integrin β1^39^. Therefore, the development of Integrin β1 inhibitors, including specific monoclonal antibodies, small molecular peptides, and mimic peptides, may become one of the effective treatments for ESCC via blocking the binding between Integrin β1 and ECM ligands.

Chemotherapy is one of the common methods for the treatment of tumors. Combined with surgery and radiotherapy, chemotherapy can significantly reduce tumor recurrence and metastasis and improve the cure rate. Focal adhesion has recently been identified as a key determinant in drug resistance^40^. As a component of focal adhesion signaling, previous studies have found that depletion of Integrin β1 enhanced the cytotoxicity of cisplatin in ESCC cells^27^. But our data show that Integrin β1 deficiency alone did not sensitize ESCC cells to cisplatin. However, when L1CAM and Integrin β1 were knocked down at the same time, the cisplatin resistance and the activation of AKT decreased significantly. These results show that L1CAM and Integrin β1 are interdependent in mediating cisplatin resistance in ESCC.

Few limitations of this study should be also acknowledged: this study only focused on the prognosis of patients with ESCC by Integrin α5β1. Several other integrin α subunits highly expressed in ESCC, such as Integrin α3, α6, α11 and αV, can also combine with Integrin β1 to form heterodimer integrins. The role of these heterodimeric integrins in the carcinogenesis of ESCC will be explored in future studies. In addition, whether the chemoresistance mediated by Integrin β1 and L1CAM is cisplatin specific, platinum specific, or applicable to other types of chemical drugs has not been studied. These problems will be explored in future research and the molecular mechanisms will be also studied in detail.

## Conclusion

By determining the expression profiling of integrins in ESCC, we identified that Integrin α5β1 was highly expressed in ESCC tissues and was associated with a poor prognosis. Integrin β1 promoted proliferation, migration, and invasion, and cooperated with L1CAM to promote chemoresistance in ESCC cells. Taken together, these results suggest the potential of Integrin α5β1 as a prognostic marker for ESCC and provide preliminary data for targeting Integrin β1 in the treatment of ESCC. However, the detailed mechanism of how Integrin β1 and L1CAM regulate tumor chemoresistance needs further study.

## Supplementary Material

Supplementary figures.

## Figures and Tables

**Table 1:** The clinicopathological characteristics of patients with ESCC

Clinical and pathological indexes		Case No.	3-year OS (%)	5-year OS (%)	P*	3-year DFS (%)	5-year DFS (%)	P*
Specimens		278						
Mean age		58						
Age (year)								
≤58		146	76.6	60.0	0.044	75.9	59.3	0.089
>58		132	72.7	56.1		72.7	54.5	
Gender								
Male		220	85.4	73.5	0.161	37.9	26.2	0.253
Female		58	39.7	0		84.5	74.0	
Tobacco use								
No		94	63.8	41.3	0.537	62.8	37.1	0.661
Yes		184	80.9	68.3		83.6	67.8	
Alcohol use								
No		195	82.0	70.1	0.550	85.1	70.1	0.738
Yes		83	56.6	39.6		56.6	38.5	
Therapies								
Preoperative Radiotherapy		2	50.0		0.832	50.0		0.713
Preoperative Chemotherapy		4	75.0		0.219	75.0		0.178
Postoperative Radiotherapy		67	46.3	32.0	0.860	46.3	31.1	0.729
Postoperative Chemotherapy		82	59.8	38.3	0.839	61.0	32.8	0.099
Tumor size								
≤3cm		62	50.9	42.7	0.114	51.3	40.7	0.045
3-5cm		132	74.1	56.5		74.8	55.7	
>5cm		84	58.3	36.9		58.3	33.3	
Tumor location								
Upper		18	0		0.270	16.7		0.105
middle		121	71.7	50.8		72.5	50.8	
Lower		139	74.8	56.8		75.5	58.3	
Histologic grade								
G1		42	47.6		0.000	47.6		0.000
G2		212	83.0	71.7		84.0	72.6	
G3		24	8.7			8.7		
Invasive depth								
T1		10	70.0		0.277	70.0		0.324
T2		46	41.5			40.1		
T3		221	84.2	75.1		87.3	72.9	
T4		1	0			0		
Lymph node metastasis								
N0		128	72.4	58.1	0.000	72.5	55.8	0.000
N1		80	57.5	43.5		55.0	38.1	
N2		52	30.8	13.9		30.8	13.5	
N3		18	5.6			0		
pTNM-stage								
I	IA	1	61.4		0.000	61.4		0.000
	IB	18						
II	IIA	54	72.4	55.1		71.6	53.6	
	IIB	70						
III	IIIA	72	74.1	59.3		74.1	58.5	
	IIIB	44						
	IIIC	19						

*Log-rank test of Kaplan Meier method; *P* <0.05 was considered significant. All patients underwent surgical treatment. OS: overall survival DFS: disease free survival

**Table 2:** The correlation between integrin α5β1 and clinicopathological characteristics in ESCC

Variables	Integrin α5β1^b^		R	chi-square value	P*
	Low	High			
Age (year)					
≤58	47	99	-0.075	1.568	0.210
>58	52	80			
Gender					
Male	16	42	-0.086	2.059	0.151
Female	83	137			
Tobacco use					
No	29	65	-0.071	1.404	0.236
Yes	70	114			
Alcohol use					
No	72	123	0.042	0.490	0.484
Yes	27	56			
Therapies^a^					
Preoperative Radiotherapy	1	1	-0.026	0.182	0.670
Preoperative Radiotherapy	1	3	0.027	0.199	0.655
Postoperative Radiotherapy	22	45	0.033	0.297	0.586
Postoperative Chemotherapy	32	50	-0.046	0.591	0.442
Tumor size					
≤3cm	20	42	-0.012	0.643	0.725
3-5cm	50	82			
>5cm	29	55			
Tumor location					
upper	6	12	0.013	0.243	0.886
middle	45	76			
lower	48	91			
Histologic grade					
G1	17	25	0.040	0.534	0.766
G2	74	138			
G3	8	16			
Invasive depth					
T1	3	7	-0.002	0.728	0.866
T2	17	29			
T3	79	142			
T4	0	1			
Lymph node metastasis					
N0	53	75	0.126	4.923	0.178
N1	28	52			
N2	13	39			
N3	5	13			
pTNM-stage					
I	8	11	0.089	2.366	0.306
II	49	75			
III	42	93			

*Fisher's Exact Test; *P* value <0.05 was considered significant. ^a^ Preoperative Radiotherapy (2 cases), Preoperative Chemotherapy (4 cases), Postoperative Radiotherapy (67 cases) and Postoperative Chemotherapy (82 cases). ^b^ low, ≤5 scores; high, >5 scores

**Table 3:** Univariate analyses and Multivariate analysis of factors associated with overall survival

Variables	Univariate analyses				Multivariate analyses			
	Sig.*	HR	95% CI for HR		Sig.*	HR	95% CI for HR	
			Lower	Upper			Lower	Upper
Age (>58 vs ≤58)	0.047	1.360	1.004	1.844	0.023	1.435	1.051	1.958
Gender (Female vs Male)	0.166	0.778	0.545	1.110				
Tobacco use (No vs Yes)	0.541	0.906	0.660	1.243				
Alcohol use (No vs Yes)	0.554	1.105	0.794	1.536				
Tumor Size	0.121							
Tumor Size (3-5cm vs ≤3cm)	0.044	0.639	0.414	0.987				
Tumor Size (>5cm vs ≤3cm)	0.202	0.80	0.568	1.127				
Tumor Location	0.279							
Tumor Location (Middle vs Upper)	0.655	1.142	0.638	2.044				
Tumor Location (Lower vs Upper)	0.164	0.797	0.578	1.098				
Histologic grade	0.000				0.002			
Histologic grade (G2 vs G1)	0.000	0.271	0.146	0.501	0.001	0.348	0.186	0.650
Histologic grade (G3 vs G1)	0.000	0.381	0.240	0.606	0.001	0.464	0.289	0.744
Invasive depth (T3+T4 vs T1+T2)	0.165	1.328	0.890	1.983				
Lymph node metastasis (N1+N2+N3 vs N0)	0.000	2.026	1.473	2.787				
Integrin α5β1	0.007	1.589	1.136	2.223	0.007	1.592	1.138	2.228

*Multivariate analysis, Cox proportional hazards regression model. Variables were adopted for their prognostic significance by univariate analysis.

**Table 4:** Univariate analyses and Multivariate analysis of factors associated with Disease free survival

Variables	Univariate analyses				Multivariate analyses			
	Sig.*	HR	95% CI for HR		Sig.*	HR	95% CI for HR	
			Lower	Upper			Lower	Upper
Age (>58 vs ≤58)	0.094	1.289	0.957	1.736				
Gender (Female vs Male)	0.260	0.818	0.576	1.160				
Tobacco use (No vs Yes)	0.665	0.934	0.685	1.274				
Alcohol use (No vs Yes)	0.741	1.056	0.764	1.461				
Tumor Size	0.050							
Tumor Size (3-5cm vs ≤3cm)	0.017	0.594	0.387	0.911				
Tumor Size (>5cm vs ≤3cm)	0.116	0.764	0.546	1.069				
Tumor Location	0.114							
Tumor Location (Middle vs Upper)	0.354	1.295	0.750	2.238				
Tumor Location (Lower vs Upper)	0.112	0.775	0.566	1.061				
Histologic grade	0.000				0.000			
Histologic grade (G2 vs G1)	0.000	0.245	0.132	0.455	0.000	0.289	0.155	0.539
Histologic grade (G3 vs G1)	0.000	0.378	0.238	0.601	0.000	0.419	0.263	0.669
Invasive depth (T3+T4 vs T1+T2)	0.253	1.258	0.849	1.864				
Lymph node metastasis (N1+N2+N3 vs N0)	0.000	2.041	1.494	2.788				
Integrin α5β1	0.011	1.524	1.101	2.108	0.014	1.504	1.086	2.083

*Multivariate analysis, Cox proportional hazards regression model. Variables were adopted for their prognostic significance by univariate analysis.

**Table 5:** IC50 of cisplatin in ESCC cell lines

Cell line	IC50 (μM)	Interval (μM)
KYSE180	2.411	2.243 to 2.591
KYSE150	2.956	2.743 to 3.186
