# Supplementary material for: Role of Integrin β1 in the progression and chemo-resistance of esophageal squamous cell carcinoma
Source: J Cancer. 2022 Mar 28;13(7):2074–85. doi: 10.7150/jca.68647 (PMC9066195; doi:10.7150/jca.68647)
Supplement: Supplementary file 1 — Supplementary figures. [file jcav13p2074s1.pdf]

Supplementary Figures

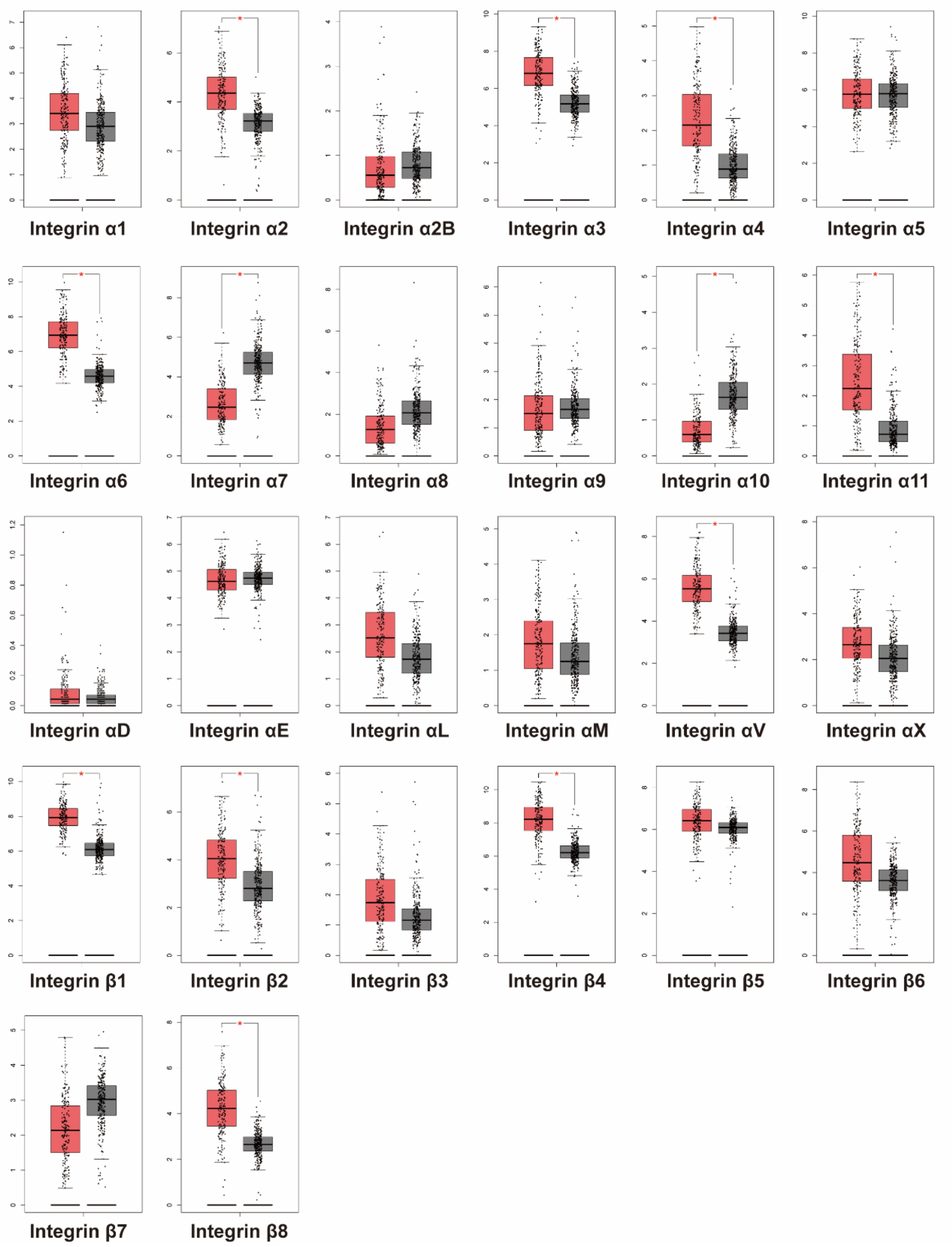

Figure S1. Expression profiling of integrins in esophageal carcinoma (data extracted from TCGA and GTEx datasets). The integrins mRNA expression profile of 182 esophageal carcinoma tissues (Red box, TCGA tumors) and 286 normal esophageal tissues (Grey box, matched TCGA normal and GTEx data) were analyzed

in Gene Expression Profiling Interactive Analysis (GEPIA). One-way ANOVA. *, $P <$ 0.05.

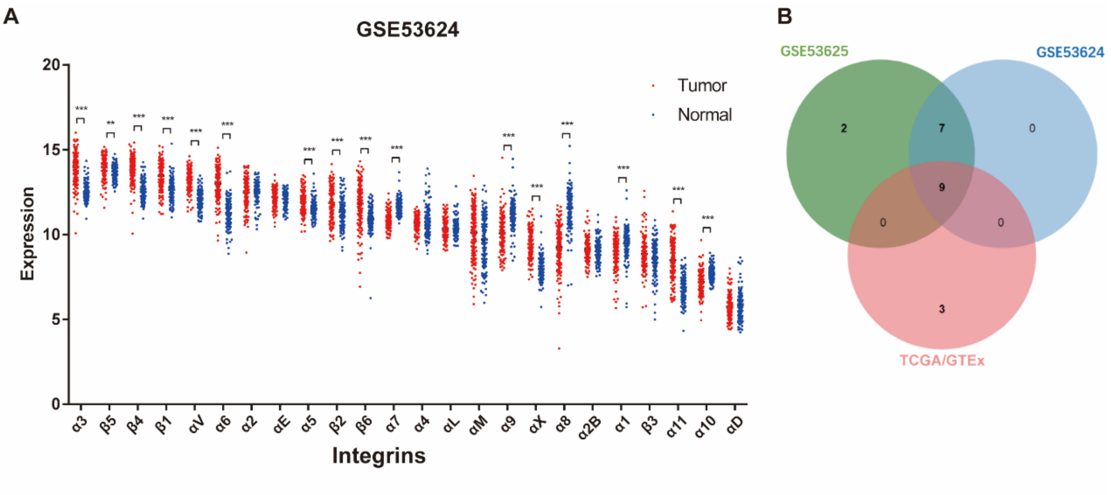

Figure S2. Expression profiling of integrins in ESCC. (A) The integrins mRNA expression profile of paired cancer and adjacent normal tissues from 119 ESCC patients (data extracted from GSE53624 dataset in GEO database). Mean ± SD. Multiple t-tests. **, $P < 0.01$, ***, $P < 0.001$. (B) Venn diagram shows the intersection of integrins differentially expressed in esophageal carcinoma and normal esophageal tissues in GSE53625, TCGA/GTEx and GSE53624 datasets.

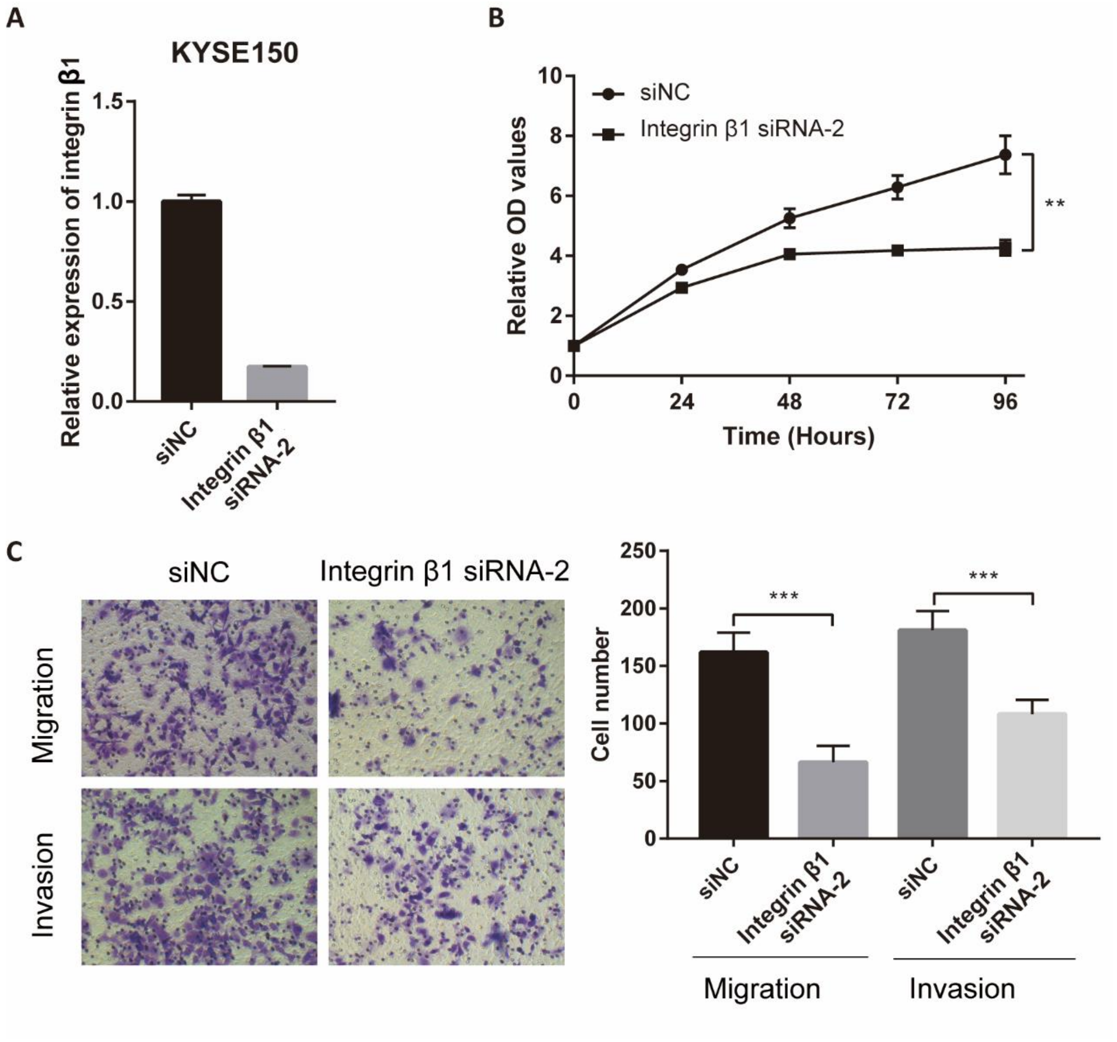

Figure S3. Integrin β1 silencing suppressed the proliferation, migration, and invasion of ESCC cells. (A) KYSE150 was transfected with negative control siRNA (siNC) or anti-Integrin β1 siRNA-2 (5'-ATCCCAGAGGCTCCAAAGATAT-3'). The knockdown of Integrin β1 was evaluated by qRT-PCR. (B) MTS assay was conducted to measure the proliferation of KYSE150 cells after Integrin β1 knockdown by siRNA. (C) Transwell Migration Assays and Transwell Invasion Assays were performed to measure the migration and invasion of KYSE150 after Integrin β1 knockdown by siRNA. The independent sample t-test was used to determine the significance of differences between groups and data was obtained in at least three independent experiments in (B) and (C). Average values are given ± SD. **, $P < 0.01$; ***, $P < 0.001$.
